# Genetic parameters and genomic prediction for feed intake recorded at the group and individual level in different production systems for growing pigs

**DOI:** 10.1186/s12711-021-00624-3

**Published:** 2021-04-08

**Authors:** Hongding Gao, Guosheng Su, Just Jensen, Per Madsen, Ole F. Christensen, Birgitte Ask, Bjarke G. Poulsen, Tage Ostersen, Bjarne Nielsen

**Affiliations:** 1 Center for Quantitative Genetics and Genomics, Aarhus University, 8830 Tjele, Denmark; 2 SEGES, Pig Research Centre, 1609 Copenhagen, Denmark

## Abstract

**Background:**

In breeding programs, recording large-scale feed intake (FI) data routinely at the individual level is costly and difficult compared with other production traits. An alternative approach could be to record FI at the group level since animals such as pigs are normally housed in groups and fed by a shared feeder. However, to date there have been few investigations about the difference between group- and individual-level FI recorded in different environments. We hypothesized that group- and individual-level FI are genetically correlated but different traits. This study, based on the experiment undertaken in purebred DanBred Landrace (L) boars, was set out to estimate the genetic variances and correlations between group- and individual-level FI using a bivariate random regression model, and to examine to what extent prediction accuracy can be improved by adding information of individual-level FI to group-level FI for animals recorded in groups. For both bivariate and univariate models, single-step genomic best linear unbiased prediction (ssGBLUP) and pedigree-based BLUP (PBLUP) were implemented and compared.

**Results:**

The variance components from group-level records and from individual-level records were similar. Heritabilities estimated from group-level FI were lower than those from individual-level FI over the test period. The estimated genetic correlations between group- and individual-level FI based on each test day were on average equal to 0.32 (SD = 0.07), and the estimated genetic correlation for the whole test period was equal to 0.23. Our results demonstrate that by adding information from individual-level FI records to group-level FI records, prediction accuracy increased by 0.018 and 0.032 compared with using group-level FI records only (bivariate vs. univariate model) for PBLUP and ssGBLUP, respectively.

**Conclusions:**

Based on the current dataset, our findings support the hypothesis that group- and individual-level FI are different traits. Thus, the differences in FI traits under these two feeding systems need to be taken into consideration in pig breeding programs. Overall, adding information from individual records can improve prediction accuracy for animals with group records.

**Supplementary Information:**

The online version contains supplementary material available at 10.1186/s12711-021-00624-3.

## Background

In animal breeding programs, most traits are usually recorded at the individual level. In pigs, traits such as feed intake (FI) and feed efficiency, which have considerable economic impact on commercial production and environmental sustainability, are costly to measure routinely at the individual level. Moreover, a characteristic of these traits is that they change with age and vary among animals of the same age but with different body weights. Thus, it is a continuing concern to obtain a sufficient amount of data to predict accurate breeding values. Since breeding for feed efficiency can be limited by the difficulty of phenotyping on a large scale in commercial herds, a cost-efficient approach needs to be considered to overcome this limitation.

In pig breeding programs, individual daily FI data are traditionally recorded at central test stations, and pigs in production farms are normally fed by electronic feeders in groups/pens of 13 to 15 boars. The latter provides the capacity to record FI data at the group level. Thus, group-level FI records might be collected on a large scale and used as an alternative or in addition to the individual-level FI records from test stations. Previous studies have demonstrated the feasibility of using group records for genetic analyses in many species, such as egg production and body weight for groups of laying hens [1–3], total FI for pens of beef cattle [4], FI for cages of mink [5, 6], and FI for groups of rabbits [7].

Recently, based on the work by Olson et al. [8], much research has been conducted on modeling group-level records, estimating variance components, and predicting genetic effects. Su et al. [9] extended the approach by Olson et al. [8] to a multifactored approach that takes non-genetic random effects, such as litter and pen effects, into account, along with the capacity of handling different group sizes. Ma et al. [10] integrated genomic information into the model by Su et al. [9], and explored a bivariate model to use information on individual-level daily gain, which is genetically correlated with FI. In their simulation study, they found that the accuracy of genetic evaluations using group records was considerably improved by adding genomic information and a genetically correlated trait recorded at the individual level for the same animals. Madsen et al. [6] used a multivariate animal model to estimate genetic (co)variances for FI, body weight, and litter size in mink, for which FI data were measured at the group (cage) level and the other two traits were measured at the individual level. In that study, instead of directly using the repeated measurements of daily FI, they defined FI as the sum of daily FI within each group.

The random regression model is commonly used for analyzing longitudinal records on individuals which are recorded along a continuous scale such as time [11, 12], and it can also be applied to longitudinal group records. This was recently demonstrated by Gao et al. [13], who analyzed longitudinal group records rather than individual-level records using a random regression model in which missing phenotypes from drop-out animals were considered over the test trajectory. Their results revealed that with group records when group composition is optimized, it is possible to achieve an accuracy comparable to the accuracy in genetic evaluation using individual records.

Feeding behaviors of individually-fed pigs at a test station may differ from feeding behaviors of pigs fed by electronic feeders in groups/pens on production farms, for example, pigs reared in groups/pens can experience competition while they are feeding, and thus may express indirect genetic effects which may be heritable. Thus, the corresponding genetic effects might differ between the two feeding systems. This implies that a possible genotype-by-environment interaction might exist for FI. In practice, the predicted breeding value for FI of breeding candidates is generally obtained based on phenotypic information from individual records at the test station, but the breeding goal is to improve feed efficiency in production farms where pigs are fed in groups. This might raise the concern of biased selection decisions due to different systems/environments.

In this study, we hypothesized that group- and individual-level FI are genetically correlated but different traits due to various factors such as different feeding systems and feeding behaviors, etc., and different environments (commercial versus central test station). Under this hypothesis, the genetic correlation between group- and individual-level FI from data recorded in these two environments is an important parameter in a pig breeding program. Thus, the aims of our study were: (1) to estimate genetic parameters and correlations between group- and individual-level FI; (2) to investigate to what extent the accuracy of prediction can be improved by adding information from individual-level FI to group-level FI to group recorded animals; and (3) to assess to what extent the accuracy of prediction can be improved by using genomic information.

## Methods

### Group-level FI data

To obtain phenotypic FI records at the group level, an experiment was conducted in a single nucleus herd (Eskegård, Haderslev, Denmark). The experiment included 6458 purebred DanBred Landrace (L) boars that were born and raised in the same nucleus herd. After weaning and growth in a weaning unit, the boars were transferred to the finishing section when their bodyweight reached about 25 kg. In the finishing section, boars were housed in pens of 10 pigs. In order to minimize variation in body weight within pens, pigs were sorted into pens by body weight. Feed was provided through a single feeder shared by two pens as described below. Hence, a feeding group consisted of two pens and a total of 323 feeding groups were recorded during the experimental period from August 2015 to October 2018. At the start of the test period, individual body weight of all the pigs in the feeding group was recorded. In the following experimental period, the total FI for each group was recorded at two time points: time point 1 and time point 2 when animals had been on test for approximately 30 and 60 days, respectively. All groups were fed ad libitum using a SpotMix feeding system (Schauer Agrotronic GmbH, Prambachkirchen, Austria). Pigs had free access to water from drinking nipples in the trough, and each feeder had a container that could hold up to 38 kg of feed; pigs in each group were continuously provided with feed. In each feeder, a sensor was mounted to avoid that the feeder ran out of feed. If the level of feed in a feeder reached the lower limit, the sensor automatically called a central feeding mixer for refilling. Each refill consisted of 15 kg feed and was weighed on a single scale in the central feeding mixer. The amount of feed was transferred from the central feeding mixer to the feeder by tubes and air pressure. The starting body weight of each animal in each pen was recorded. To obtain the amount of feed intake of the pigs in each feeding group during each period between body weight records, the sum of feed delivered to the feeding group was adjusted by the amount of feed in the container of the feeder at the beginning and end of each given time period.

**Fig. 1:**
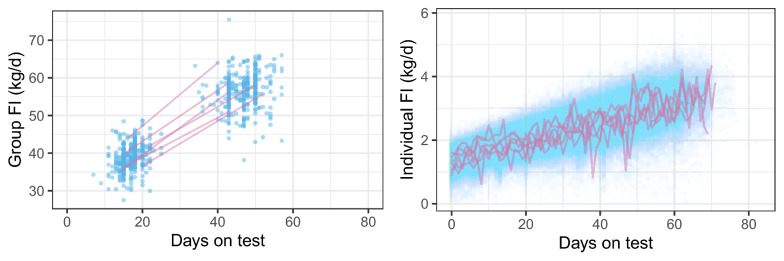
Daily feed intake (kg/d) over days on test at the group level (left) and individual level (right). The lines represent the trajectories of six randomly sampled groups/animals

During the experiment, 99 boars were removed from the groups, which were referred as drop-out animals due to sickness or death. The average daily FI at the group level were used as the phenotype, which was calculated as the total FI of the group divided by the number of days during which animals ate in each group. A mid-point date in the test period was used as the testing date for the average daily FI. Figure 1 (left panel) shows the average daily group-level FI over days on test.

### Individual-level FI data

Data on individual daily FI that were recorded on purebred DanBred Landrace boars at the central test station (Bøgildgård, Kjellerup, Denmark) were supplied by SEGES, Pig Research Centre, Denmark. All tested boars were born in nucleus herds and were kept at the test station while their body weight was between ~ 30 to 100 kg. Boars were housed in pens of 10 to 12  pigs and fed ad libitum by BIAS Schauer feeders (Schauer Agrotronic GmbH, Prambachkirchen, Austria) during the whole test period. The pigs could access the feeder continuously day and night, and thus the feeders were not overstretched during the experiment, which further minimized competition between pigs while they were eating. During the test period and for each boar, individual FI was automatically recorded each time it visited the electronic feeder, and the daily FI was calculated as the sum of all FI records during a day. The final daily FI records were retrieved for the period from August 2015 to February 2019.

In total, 271,001 daily feed intake observations from 4526 boars were available for the analyses and Fig. 1 (right panel) presents the individual daily FI over days on test.

### Pedigree and genotype information

Animals for which either a group-level FI record or individual-level FI records were available were traced back to year 1988, and the final pedigree included 19,734 animals. In total, 14,791 animals were genotyped using the 50 K NEOGEN GeneSeek Genomic Profiler (GGP) Porcine BeadChip (GeneSeek, Lincoln, NE) and 10,054 of them had records (either group-level or individual-level). All the genotyped animals and animals with records were included in the pedigree. Table 1 presents an overview of the numbers of animals. In total, 37,621 SNPs across the 18 pig autosomes met the following requirements: (1) each SNP had a minor allele frequency (MAF) higher than 0.01, (2) had a call rate score greater than 0.9, (3) showed no strong deviation from Hardy–Weinberg equilibrium (P > 10^−7^ for a Chi square test), and (4) had a known position on the porcine Build 10.2 assembly [14]. Moreover, for each animal and SNP-genotype combination, if the GenCall score was less than 0.6, genotypes were defined as missing and animals for which the average call rate was less than 0.8 were excluded from the analysis. Genotypes were imputed with the FImpute v 2.2 software [15].

**Table 1:** Number of genotyped animals with records, number of non-genotyped animals with records, number of records, mean and standard deviation (SD) of group- and individual-level feed intake (FI) over the test period and for different days on test (DOT) classes

DOT class^a^	Number of animals with records^c^		Number of records	Mean	SD
	Genotyped	Non-genotyped			
Group FI^b^	6047	348	646	47.77	9.782
~ 30	6047	348	323	38.92	3.483
~ 60	5960	336	323	56.63	4.710
Individual FI	3962	564	271,001	2.28	0.704
$\le$10	3962	564	49,472	1.52	0.327
11–20	3962	563	44,971	1.86	0.352
21–30	3944	556	44,713	2.16	0.397
31–40	3929	551	44,341	2.43	0.468
41–50	3881	530	42,695	2.73	0.537
51–60	3423	458	32,669	3.04	0.583
61–70	2255	315	11,651	3.25	0.659
$\ge$ 71	86	11	489	3.43	0.674

^a^For group-level FI, ~ 30 and ~ 60 represent two recording time points at which animals have been approximately on test during 30 and 60 days, respectively

^b^Average group size = 19.6

^c^Animals with group-level records were also considered

### Statistical models

A bivariate random regression model that treated group- and individual-level FI as different traits was used. In addition to this, a univariate model based only on group-level FI data was applied to evaluate whether adding the extra information from individual-level FI could improve the predictive ability of the model.

Heterogeneity of residual variance was initially investigated for both group- and individual-level FI records based on univariate models. To account for heterogeneous residual variances, the phenotypes were allocated to different classes based on days on test, assuming homogeneity within classes and heterogeneity between classes. The group-level FI records were divided into two test classes, 1 and 2 (based on two recording time points), and the individual FI records were divided into eight test classes from 3 to 10 (< 10, 11–20, 21–30, 31–40, 41–50, 51–60, 61–70, > 71 days on test).

Nested models (i.e., a model that assumes heterogeneous residual variance and a model that assumes homogeneous residual variance) were compared using the likelihood-ratio test [16, 17], which suggested that the use of homogeneous residual variance was appropriate for group-level FI records, whereas the use of heterogeneous residual variance was appropriate for individual-level FI records (results not shown).

#### Bivariate model

The bivariate random regression model for joint analysis of individual-level and group-level FI was as follows:

1 $${y}_{jltm}={YM}_{l}+\sum_{k=0}^{1}\stackrel{-}{{w}_{j}}{\beta }_{k\left(m\right)}+\sum_{k=0}^{nf}{\varnothing \left(t\right)}_{jk}{b}_{k\left(j*m\right)}+\sum_{i=1}^{{n}_{jt}}\sum_{k=0}^{nr}{\varnothing (t)}_{ijk}{a}_{ijk}+\sum_{i=1}^{{n}_{jt}}\sum_{k=0}^{np}{\varnothing (t)}_{ijk}{pe}_{ijk}+{e}_{jltm},$$

2 $${y}_{iltm}={YM}_{l}+\sum_{k=0}^{1}{w}_{i}{\beta }_{k\left(m\right)}+\sum_{k=0}^{nf}{\varnothing (t)}_{ik}{b}_{k}+\sum_{k=0}^{nr}{\varnothing (t)}_{ik}{a}_{ik}+\sum_{k=0}^{np}{\varnothing (t)}_{ik}{pe}_{ik}+{e}_{iltm},$$

where in Eq. (1), ${y}_{jltm}$ is the daily FI of group $j$ measured at time point $t$ within year-month (YM) $l$ and belonging to test class $m$, $m\in \left\{1, 2\right\}$; $YM$ represents the fixed effect of the start year-month of the experiment for group $j$; $\stackrel{-}{{w}_{j}}$ is the covariate of mean start body weight for animals in group $j$; ${\beta }_{k(m)}$ denotes the $k$th regression coefficient nested within days on test class $m$; ${b}_{k}$ is the $k$th fixed regression coefficient nested within the combination of group size of group $j$, group size $\in \left\{14, 15, 16, 17, 18, 19, 20\right\}$ and test class $m$, i.e., ${j}^{*}m$; ${a}_{ijk}$ and ${pe}_{ijk}$ are the $k$th random regression for the additive genetic and permanent environmental effects, respectively, of animal $i$ in group $j$; ${\varnothing (t)}_{ijk}$ is the time covariate defined by the $k$th Legendre polynomial at time point $t$ for animal $i$ in group $j$, and the intercept of ${\varnothing (t)}_{ijk}$ (when $k=0$) was standardized to 1; ${n}_{jt}$ is the number of animals in group $j$ at time point $t$; ${e}_{jltm}$ is the random residual; and in Eq. (2), ${y}_{iltm}$ is the individual daily FI of animal $i$ measured at time $t$ within year-month (YM) $l$; $YM$ is the fixed effect, representing the start year-month of animal $i$; ${w}_{i}$ is the covariate of start body weight for animal $i$; ${\beta }_{k(m)}$ denotes the $k$th regression coefficient nested within test class $m$, $m\in \{3, 4, 5, 6, 7, 8, 9, 10\}$; ${b}_{k}$ is the $k$th fixed regression coefficient; ${a}_{ik}$ and ${pe}_{ik}$ are the $k$th random regression of additive genetic and permanent environmental effects for animal $i$, respectively; ${\varnothing (t)}_{ik}$ is the time covariate defined by the $k$th Legendre polynomial at time point $t$ for animal $i$; $nf$, $nr$, and $np$ are the orders of Legendre polynomials fitted for the fixed regression, genetic, and permanent environmental effects, respectively, ($nf\ge \mathrm{max}(nr,np)$); and ${e}_{iltm}$ is the residual.

Models with different orders of Legendre polynomials were applied and compared using the Akaike information criterion (AIC) [18] and Bayesian information criterion (BIC) [19] (results not shown). Based on this investigation, first order Legendre polynomials were fitted for group records, while second order Legendre polynomials were fitted for individual records. Therefore, the distribution of the regression coefficients of the genetic effects was:

$$\left[ {{\mathbf{a}}_{0}^{g} \;{\mathbf{a}}_{1}^{g} \;{\mathbf{a}}_{0}^{i} \;{\mathbf{a}}_{1}^{i} \;{\mathbf{a}}_{2}^{i} } \right]^{\prime } \;\sim \;MVN\left( {{\mathbf{0}},{\mathbf{G}} \otimes {\mathbf{K}}} \right),$$

where superscripts denote the type of record (“$g$” for group-level FI and “$i$” for individual-level FI) and indices 0 and 1 refer to intercept and slope, respectively; $\mathbf{G}$ is a 5 × 5 (co)variance matrix between these five additive genetic random coefficients; $\mathbf{K}$ is the genetic relationship matrix, referring to the numerator relationship matrix $\mathbf{A}$ for pedigree-based best linear unbiased prediction (PBLUP), or the combined relationship matrix $\mathbf{H}$ for single-step genomic best linear unbiased prediction (ssGBLUP); ⨂ is the Kronecker product.

The distribution of coefficients for permanent environmental effects was:

$$\left[ {{\mathbf{pe}}_{0}^{g} \;{\mathbf{pe}}_{1}^{g} \;{\mathbf{pe}}_{0}^{i} \;{\mathbf{pe}}_{1}^{i} \;{\mathbf{pe}}_{2}^{i} } \right]^{\prime } \sim MVN\;\left( {{\mathbf{0}},{\mathbf{P}}} \right),$$

where $${\mathbf{P}} = \left[ {\begin{array}{*{20}c} {{\mathbf{I}}_{p} \otimes {\mathbf{P}}_{1} } & {\mathbf{0}} \\ {\mathbf{0}} & {{\mathbf{I}}_{q} \otimes {\mathbf{P}}_{2} } \\ \end{array} } \right]$$, and ${\mathbf{P}}_{1}$ represents a 2 × 2 (co)variance matrix between the two permanent environmental random regression coefficients for animals with group records; ${\mathbf{I}}_{p}$ is an identity matrix of order $p$, corresponding to the number of groups with phenotypes; ${\mathbf{P}}_{2}$ represents a 3 × 3 (co)variance matrix between the three permanent environmental random regression coefficients for animals with individual records; ${\mathbf{I}}_{q}$ is an identity matrix of order $q$, corresponding to the number of individuals with phenotypes.

The residuals $$\left[ {{\mathbf{e}}^{g} {\mathbf{e}}^{i} } \right]^{\prime } \sim MVN\left( {{\mathbf{0}},{\mathbf{R}}} \right),$$

with

$${\mathbf{R}} = \left[ {\begin{array}{*{20}c} {{\mathbf{D}}_{n} \sigma _{e}^{2} } & {} & {} & {} \\ {} & {{\mathbf{I}}_{{n1}} \sigma _{{e1}}^{2} } & {} & {} \\ {} & {} & \ddots & {} \\ {} & {\mathbf{0}} & {} & {{\mathbf{I}}_{{n8}} \sigma _{{e8}}^{2} } \\ \end{array} } \right],$$

where $\sigma_{e}^{2}$ is the residual variance for group FI records, ${\mathbf{D}}_{n}$ is a diagonal matrix for group records with $n$ equal to the total number of group records and the diagonal elements equal to $nt/nd$, where $nt$ is the number of animals in a group at the time point $t$, and $nd$ is the length (in days) of the period; $\sigma_{e1}^{2}$ to $\sigma_{e8}^{2}$ are the residual variances for individual-level FI, and ${\mathbf{I}}$ is the identity matrix. The subscripts $n1$ to $n8$ denote the numbers of phenotypic records in the eight classes of days on test.

#### Univariate model

The group-level FI data were also analyzed independently with a univariate model. The univariate model is a sub-model of the bivariate model in Eq. (1). The random regression coefficients of the genetic and permanent environmental effects were assumed to be respectively:

$$\left[ {{\mathbf{a}}_{0}^{g} \;{\mathbf{a}}_{1}^{g} } \right]^{\prime } \sim MVN\left( {\mathbf{0},{\mathbf{K}} \otimes {\mathbf{G}}} \right),$$

$$\left[ {{\mathbf{pe}}_{0}^{g} \;{\mathbf{pe}}_{1}^{g} } \right]^{\prime } \sim MVN\;\left( {{\mathbf{0}},{\mathbf{P}} \otimes {\mathbf{I}}} \right),$$

and the residual effects follow $N\left( {\mathbf{0},{\mathbf{I}}\sigma_{e}^{2} } \right),$ where ${\mathbf{G}}$ is a 2 × 2 (co)variance matrix between two additive genetic random regression coefficients; ${\mathbf{P}}$ is a 2 × 2 (co)variance matrix between two permanent environmental random regression coefficients, ${\mathbf{K}}$ is the relationship matrix referring to ${\mathbf{A}}$, the pedigree-based relationship matrix used in PBLUP, or to ${\mathbf{H}}$, the combined pedigree- and genomic-based relationship matrices used in ssGBLUP, and ${\mathbf{I}}$ is the identity matrix.

### Estimates of variance component and heritability

Variance components were estimated by restricted maximum likelihood (REML) [20] using the average information REML (AI-REML) algorithm [21–23]. Breeding values were predicted by both PBLUP and ssGBLUP [24]. All analyses were performed using the DMU package [25].

The genetic and permanent environmental variances were calculated using the (co)variance function in Eq. (3) for each day within the test period. Additive genetic variance $\sigma_{at}^{2}$ for day $t$ during the test period was calculated as follows:

3 $$\sigma _{{at}}^{2} = {\boldsymbol{\upphi }}_{{\text{t}}} {\mathbf{G}}{\boldsymbol{\upphi }}_{{\text{t}}}^{\prime } ,$$

where ${\boldsymbol{\upphi }}_{t}$ is a row vector of the time covariate defined by the Legendre polynomial on day $t$, ${\mathbf{G}}$ is (co)variance matrix between additive genetic random regression coefficients. The same calculation was conducted for the permanent environmental variance ($\sigma_{pet}^{2}$). The estimates of phenotypic variance were defined as the sum of the estimates of genetic, permanent environmental, and residual variances for each day during the test period, i.e., $\sigma_{pt}^{2} = \sigma_{at}^{2} + \sigma_{pet}^{2} + \sigma_{et}^{2}$, where $\sigma_{et}^{2}$ is the residual variance for day $t$. The estimates of heritability for each day during the test period was calculated as $\sigma_{at}^{2} /\sigma_{pt}^{2}$.

### Estimation of the genetic correlation

The genetic correlation between group- and individual-level FI for each test day $t$ was computed as follows: $r_{t} = \frac{{{\boldsymbol{\upphi }}_{{{\text{t}}2}} {\mathbf{G}}_{12} {\boldsymbol{\upphi }}_{{{\text{t}}1}}^{^{\prime}} }}{{\sqrt {{\boldsymbol{\upphi }}_{{{\text{t}}1}} {\mathbf{G}}_{1} {\boldsymbol{\upphi }}_{{{\text{t}}1}}^{^{\prime}} } \sqrt {{\boldsymbol{\upphi }}_{{{\text{t}}2}} {\mathbf{G}}_{2} {\boldsymbol{\upphi }}_{{{\text{t}}2}}^{^{\prime}} } }}$, where ${\boldsymbol{\upphi }}_{t1}$ and ${\boldsymbol{\upphi }}_{t2}$ are the row vectors of the time covariate defined by the Legendre polynomial on day $t$ for group and individual records, respectively; ${\mathbf{G}}_{12}$, ${\mathbf{G}}_{1}$ and ${\mathbf{G}}_{2}$ are the sub-matrices of ${\mathbf{G}}$; and subscripts 1 and 2 denote group- and individual-level FI. The genetic correlation between group- and individual-level FI during the whole test period was computed as $r = \frac{{{\boldsymbol{\upphi }}_{2}^{\varvec{*}} {\mathbf{G}}_{12} {\boldsymbol{\upphi }}_{1}^{{\varvec{*^{\prime}}}} }}{{\sqrt {{\boldsymbol{\upphi }}_{1}^{\varvec{*}} {\mathbf{G}}_{1} {\boldsymbol{\upphi }}_{1}^{{\varvec{*^{\prime}}}} )} \sqrt {{\boldsymbol{\upphi }}_{2}^{\varvec{*}} {\mathbf{G}}_{2} {\boldsymbol{\upphi }}_{2}^{{\varvec{*^{\prime}}}} )} }}$, where ${\boldsymbol{\upphi }}_{1}^{*}$ (two elements) and ${\boldsymbol{\upphi }}_{2}^{*}$ (three elements) are row vectors derived from ${\boldsymbol{\upphi }}_{1}$ and ${\boldsymbol{\upphi }}_{2}$, respectively by summing the column-wise elements, i.e., $${\boldsymbol{\upphi }}_{1}^{*} = \left[ {\begin{array}{*{20}c} {\mathop \sum \nolimits_{i = 1}^{n} {{\boldsymbol{\upphi }}}_{i1} } {\mathop \sum \nolimits_{i = 1}^{n} {{\boldsymbol{\upphi} }}_{i2} } \\ \end{array} } \right]$$ and $${\boldsymbol{\upphi }} _{2}^{*} = \left[ {\begin{array}{*{20}c} {\sum\nolimits_{{i = 1}}^{n} {{{\boldsymbol{\upphi} }}_{{i1}} } } {\sum\nolimits_{{i = 1}}^{n} {{{\boldsymbol{\upphi} }}_{{i2}} } } {\sum\nolimits_{{i = 1}}^{n} {{{\boldsymbol{\upphi} }} _{{i3}} } } \\ \end{array} } \right]$$, where $n$ is equal to the number of test days during the test period.

### Validation of the models

A leave-one-group-out cross-validation strategy was used based on animals with group-level FI records, i.e., each group in the group-level FI dataset was left out in turn and the remaining dataset is referred to as the reduced dataset. Accordingly, the original dataset without leaving out any group is referred to as the full dataset. Genetic prediction was conducted based on all the reduced datasets with pre-estimated variance components based on the corresponding full dataset using matrix ${\mathbf{A}}$ as the genetic covariance structure. The estimated breeding values were termed as EBV and GEBV when using PBLUP and ssGBLUP, respectively. The (G)EBV of a given animal $i$ on a given test day $t$ was calculated as $\left( G \right)EBV_{it} = {\boldsymbol{\upphi }}_{it} {\mathbf{a}}_{i}$, where ${\mathbf{a}}_{i}$ is a column vector of length 2, containing regression coefficients of the genetic effects for animal $i$, ${\boldsymbol{\upphi }}_{it}$ is a row vector of length 2, containing the time covariate defined by the Legendre polynomial on day $t$ for animal $i$. The final (G)EBV used for validation were computed as the sum of the (G)EBV for each animal over the test days when group-level FI were recorded.

To validate and compare the quality of prediction between models, we used the four statistics as presented in Legarra and Reverter [26]: (1) the Pearson’s correlation between (G)EBV based on full and reduced data ($\rho_{f,r}$); (2) the slope of the regression of (G)EBV obtained from full data on (G)EBV obtained from reduced data ($b_{f,r}$); (3) the Pearson’s correlation between (G)EBV based on reduced data and corrected phenotypes ($\rho_{{y_{c} ,r}}$); and (4) the slope of the regression of corrected phenotypes on (G)EBV obtained from reduced data ($b_{{y_{c} ,r}}$). For (1) and (2), the statistics were calculated separately for all the animals in the group-level FI dataset; the subset of genotyped animals in the group-level FI dataset; and the subset of non-genotyped animals in the group-level FI dataset. For (3) and (4), the corrected phenotypes ($y_{c}$) were computed based on the full data of group-level FI by correcting the phenotype for fixed effects and non-genetic random effects of all the animals in the group, i.e., $y_{c} = \mathop \sum \nolimits_{i = 1}^{n} \left( G \right)EBV_{i} + \hat{e}$, where $n$ is the number of animals in the group.

## Results

### Variance components and heritability

Given that the group-level FI records were analyzed with the traditional linear mixed model but with appropriate modifications regarding incidence matrices and residuals, the estimated variance components from group records were on the same scale as those from individual records. Therefore, it should be noted that these variance components and heritabilities are comparable, and are expected to be close if group- and individual-level FI are the same trait [9, 13].

**Fig. 2:**
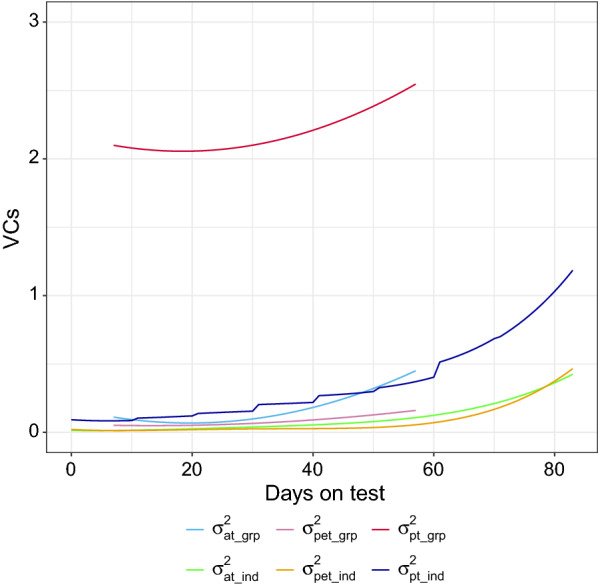
Trajectory of estimated additive genetic variance ($\sigma_{at}^{2}$), permanent environmental variance ($\sigma_{pet}^{2}$), and phenotypic variance ($\sigma_{pt}^{2}$) over days on test for group- (grp) and individual-level (ind) feed intake data from the pedigree-based bivariate model using the full dataset

Figure 2 shows the estimated additive genetic variance, permanent environmental variance, and phenotypic variance as a function of days on test for group- and individual-level FI. The estimated genetic variances for group-level FI were on average 0.17 (SD = 0.11), and the estimated genetic variances for individual-level FI were on average 0.10 (SD = 0.11). The estimated permanent environmental variances for group-level FI were on average 0.08 (SD = 0.03), and the estimated permanent environmental variances for individual-level FI were on average 0.08 (SD = 0.11). The estimates of the phenotypic variance for group-level FI were on average 2.19 (SD = 0.15), and the estimates of the phenotypic variance for individual-level FI were on average 0.36 (SD = 0.29). The much higher estimates of the phenotypic variance for group-level FI resulted from the higher estimated residual variance, i.e., 1.94, whereas the average estimate of the residual variance for individual-level FI was 0.17 (SD = 0.10). For both group- and individual-level FI, larger genetic variances were observed during the end of the test period. However, the curves of the estimated genetic variances for group-level FI showed a sharper increase from the middle to the end of the test period than those for individual-level FI. The curves of the phenotypic variance and genetic variances presented similar patterns.

**Fig. 3:**
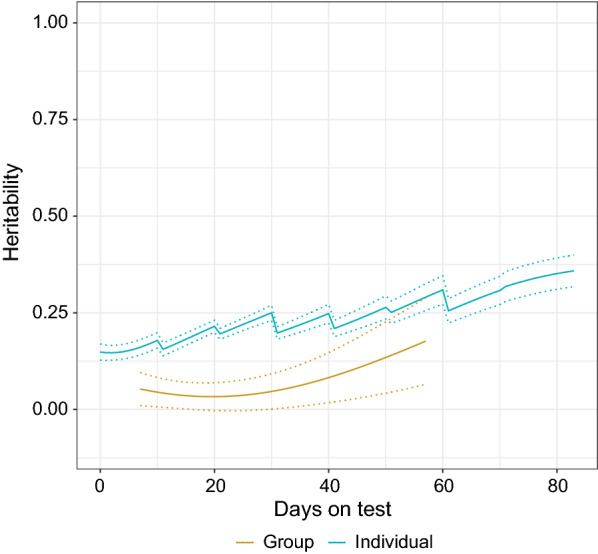
Trajectory of estimated heritability (solid line) and heritability ± standard error (SE) (dotted line) over days on test for group- and individual-level feed intake data from the pedigree-based bivariate model using the full dataset

Figure 3 shows the trajectories of the estimated heritabilities and their standard errors (SE) over days on test for group- and individual-level FI. The estimates of heritability ranged from 0.03 to 0.18 (average 0.07) for group-level FI, and from 0.15 to 0.36 (average 0.24) for individual-level FI over the test period. The SE of the heritability estimates were much larger for group-level FI than those for individual-level FI (Fig. 3). Heritabilities for both group- and individual-level FI were not constant throughout the days on test but tended towards higher values during the late stages of the test period, whereas a sharper upward trajectory was observed for group-level FI compared with individual-level FI.

### Genetic correlations

**Fig. 4:**
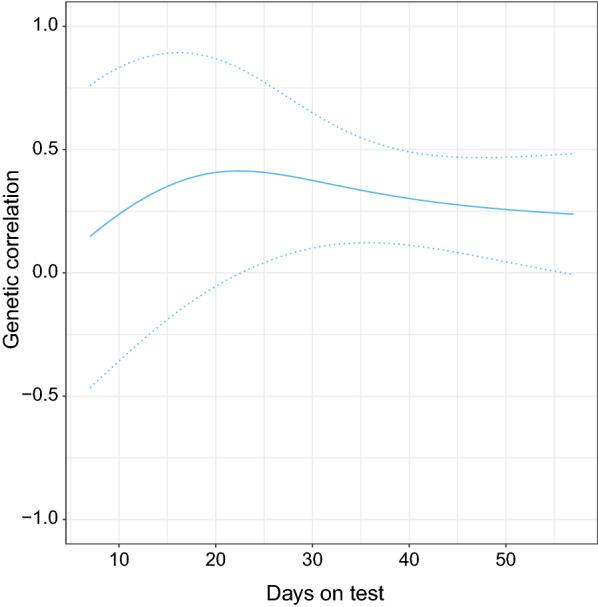
Trajectory of estimated genetic correlations (solid line) between group- and individual-level feed intake, and genetic correlations ± standard error (SE) (dotted line) over days on test from the pedigree-based bivariate model using the full dataset

Figure 4 displays the trajectory of the estimated genetic correlations between group- and individual-level FI over days on test. The estimated genetic correlations were on average 0.32 (SD = 0.07) over the test period. Overall, the curve remained stable although an initial increase can be observed during the very early stage of the test period. The SE were large especially at the beginning of the test period. A genetic correlation of 0.23 was estimated between FI summed over the test period for group-level and for individual-level FI.

### Accuracy of prediction

Table 2 shows the Pearson’s correlations between (G)EBV based on the full and reduced datasets ($\rho_{f,r}$). Compared to using only group-level FI records (bivariate vs. univariate model), adding information from individual-level FI records to group-level FI records increased the correlation by 0.013 for both PBLUP and ssGBLUP. Furthermore, adding genomic information increased the correlation by 0.048 (ssGBLUP vs. PBLUP) for both univariate and bivariate models. The slopes of the regression of (G)EBV obtained with the full dataset on (G)EBV obtained with the reduced dataset ($b_{f,r}$) were close to unity for genotyped animals, whereas a slight over-dispersion of the EBV from the reduced data compared to those from the full data was observed for non-genotyped animals when using ssGBLUP (Table 2). Additional file 1: Figure S1 provides a scatter plot of the Pearson’s correlations of (G)EBV based on the full and reduced datasets ($\rho_{f,r}$) versus the absolute difference in (G)EBV between the full and reduced datasets ($d_{f,r}$) for all 323 groups for the univariate PBLUP (panel A), bivariate PBLUP (panel B), univariate ssGBLUP (panel C), and bivariate ssGBLUP (panel D).

**Table 2:** Pearson’s correlations of (genomic) estimated breeding values (G)EBV based on full and reduced datasets ($\rho_{f,r}$), and slope (SE) of the regression of (G)EBV obtained from the full dataset on (G)EBV obtained from the reduced dataset ($b_{f,r}$) using univariate pedigree-based best linear unbiased prediction (PBLUP), univariate single-step genomic best linear unbiased prediction (ssGBLUP), the bivariate PBLUP, or bivariate ssGBLUP model

	All		Genotyped		Non-genotyped	
	${\varvec{\rho}}_{{{\varvec{f}},{\varvec{r}}}}$	${\varvec{b}}_{{{\varvec{f}},{\varvec{r}}}}$	${\varvec{\rho}}_{{{\varvec{f}},{\varvec{r}}}}$	${\varvec{b}}_{{{\varvec{f}},{\varvec{r}}}}$	${\varvec{\rho}}_{{{\varvec{f}},{\varvec{r}}}}$	${\varvec{b}}_{{{\varvec{f}},{\varvec{r}}}}$
Univariate^a^						
PBLUP	0.890	1.00 (0.006)	0.890	1.00 (0.007)	0.886	0.97 (0.027)
ssGBLUP	0.938	0.99 (0.005)	0.939	0.99 (0.005)	0.904	0.95 (0.024)
Bivariate^b^						
PBLUP	0.903	1.00 (0.006)	0.903	1.00 (0.006)	0.908	0.99 (0.025)
ssGBLUP	0.951	0.98 (0.004)	0.952	0.98 (0.004)	0.921	0.96 (0.022)

$\rho_{f,r}$ and $b_{f,r}$ were calculated separately based on all animals in the group-level dataset, the subset of genotyped animals in the group-level data set, and the subset of non-genotyped animals in the group-level dataset

^a^Univariate analyses were based on the group-level dataset only

^b^Bivariate analyses were based on the combination of group- and individual-level datasets

Table 3 presents the Pearson’s correlations between group (G)EBV calculated as the sum of (G)EBV for all animals in the group and the corrected phenotypes of group-level FI ($\rho_{{y_{c} ,r}}$). This correlation can be used to evaluate the prediction accuracy. Adding information from individual-level FI records to group-level FI records, increased the prediction accuracy by 0.018 and 0.032 compared to using group-level FI records (bivariate vs. univariate model) for PBLUP and ssGBLUP, respectively. Furthermore, adding genomic information increased the prediction accuracy by 0.019 and 0.033 (ssGBLUP vs. PBLUP) for the univariate and bivariate models, respectively. A slight over-dispersion ($b_{{y_{c} ,r}}$) was observed when using the ssGBLUP model (Table 3).

**Table 3:** Pearson’s correlations between group (genomic) estimated breeding values ((G)EBV) calculated as the sum of (G)EBV for animals in each group and the corrected phenotypes of group-level feed intake ($\rho_{{y_{c} ,r}}$), and slope (SE) of the regression of corrected phenotypes of group-level feed intake on group (G)EBV ($b_{{y_{c} ,r}}$) calculated as the sum of (G)EBV for animals in each group from univariate pedigree-based best linear unbiased prediction (PBLUP) and single-step genomic best linear unbiased prediction (ssGBLUP), the bivariate PBLUP and ssGBLUP models

	All 323 groups	
	${\varvec{\rho}}_{{{\varvec{y}}_{{\varvec{c}}} ,{\varvec{r}}}}$	${\varvec{b}}_{{{\varvec{y}}_{{\varvec{c}}} ,{\varvec{r}}}}$
Univariate^a^		
PBLUP	0.578	1.00 (0.079)
ssGBLUP	0.597	0.91 (0.068)
Bivariate^b^		
PBLUP	0.596	1.00 (0.075)
ssGBLUP	0.629	0.87 (0.060)

^a^Univariate analyses were based on the group-level dataset only

^b^Bivariate analyses were based on the combination of group- and individual-level datasets

## Discussion

In our study, we addressed the hypothesis that group- and individual-level FI are different traits for pigs recorded in groups at the nucleus herd versus pigs individually recorded at the test station. We used a bivariate random regression model to estimate variance components and genetic correlations. Through an appropriate modification of the traditional linear mixed model, the variance components estimated from group-level FI were on the same scale as those estimated from individual-level FI. The results support the hypothesis that group- and individual-level FI are genetically correlated but different traits.

### Heritability of feed intake during the growth period

The trajectories in Fig. 3 show that the estimates of heritability for group-level FI were significantly lower than those for individual-level FI. This outcome contradicts previously published studies that analyzed group records using univariate models. Biscarini et al. [3] analyzed cage records of early egg production based on real data in laying hens, and found that the estimated heritability did not deviate from the heritability for individual records. Peeters et al. [1] showed that the estimated variance components from pooled records did not significantly differ from the individual records when analyzing traits affected by social interactions in laying hens. In a simulation study, Su et al. [9] mimicked the FI trait in pigs, and developed a model that could handle single-group records by taking varying group sizes and extra non-genetic random effects (litter and pen effects) into account for the estimation of variance components and genetic prediction. Their results demonstrated that variances estimated from group records were consistent with those from individual records but with higher SE. More recently, Gao et al. [13] further extended the approach of Su et al. [9], to analyze longitudinal group records of FI. Their results showed that, generally, the use of group records yielded similar estimates of variance components compared to that of individual records but with larger SE.

The large estimates of the residual variance from group-level FI that we observed here could be caused by the fact that the model ignores the random pen effect as reported by Su et al. [9]. In their study, they found that the residual variance increased from 209 to 558 after removing the pen effect in the model when analyzing the group records, and that ignoring the pen effect has less influence on the estimated residual variance when using individual records. This indicates that a large part of the pen effects moved to the residuals. However, given that, in our study, the number of animals across pens was nearly constant, it was difficult to separate the pen effects from the residual effects since the covariance matrix of the pen effects cannot be distinguished from the residual covariance matrix when group size is (nearly) constant [9].

To further examine the pattern of the larger estimated residual variance that was obtained by analyzing the group-level FI data, a similar analysis was performed by converting the current individual-level data to the corresponding group-level structure. We constructed two test periods similar to those used for the group records, summed up the individual records of boars from the same pen over animals and days within the period, and applied to these group records the same univariate model as that used for group-level FI data. We found a much larger estimated residual variance than that obtained from the individual-level data. This test indicates that another reason for the large estimates of residual variance obtained from group-level FI could be that our current model assumes that group-level records are summed up over both animals and days during each period instead of being summed only over animals. However, it is unlikely that all the residuals are uncorrelated, and in addition, our current model ignores this effect and assumes that residual effects are independent. Moreover, the total number of groups included in the analysis (323) was relatively small (also for the test above). Therefore, with such limited information available, the estimates of the variance component and heritability might be unprecise for the group-level FI.

Estimates of heritability for individual-level FI in pigs have been well documented in previous studies. Schnyder et al. [27] reported heritabilities of 0.09 to 0.25 for daily FI using a random regression model with second-order Legendre polynomial in French Landrace and Large White pigs. By using a model similar to Schnyder et al. [27], Cai et al. [28] found heritabilities of daily FI that ranged from 0.10 to 0.37 in Yorkshire boars. Shirali et al. [29] developed a horizontal model for a combined analysis of longitudinal FI and single recorded production traits, and showed that the estimated heritabilities of FI ranged from 0.13 to 0.22 in Landrace pigs. Our estimates of heritability for individual-level FI are generally in line with these findings.

### Genetic correlation between group- and individual-level FI

In practice, group-level FI data can be measured in the nucleus breeding herds, whereas individual-level FI data are usually measured at a central test station using a different feeding system that may result in different feeding behaviors of the pigs. In addition, the two environments may have different management systems. It is somewhat surprising that very low genetic correlations between group and individual FI were observed (significantly different from 1) over the whole test period in our study (Fig. 4). This indicates that considerable genotype-by-environment interaction between these two environments exists, and thus FI recorded under these two environments are clearly different traits.

### Effect of the bivariate vs. univariate model on genetic/genomic prediction

Accuracy of predicted breeding values ($\rho_{f,r}$) and prediction accuracy ($\rho_{{y_{c} ,r}}$) were both improved when information from individual-level FI records were added to group-level FI records via the bivariate model in comparison to the univariate model with group-level FI records only. Ma et al. [10] reported similar results from a simulation study in which two traits (e.g. feed intake and daily gain) with group records for trait one and individual records for trait two were simulated based on a genetic correlation of 0.8. Their result showed that the gain in prediction accuracy from the bivariate model for the group recorded trait (trait one) was 11 to 22 percentage points compared with the univariate model.

### Other factors impacting prediction accuracy

Group size is known to be a crucial factor when using group records for prediction of breeding values. The use of records from large groups tends to reduce prediction accuracy compared to that from small groups [8–10]. In our study, the size of the group was relatively large, i.e., 20, since one feeder was shared by two pens. Therefore, with a smaller group size and with close genetic relationships between group members, prediction accuracy might be further improved for group recorded animals.

In addition to the direct genetic effect (DGE), the indirect genetic effect (IGE) is also a well-known heritable effect caused by group mates [30–32]. In pig breeding, traits such as FI and average daily gain (ADG) may be subject to competition among group members, and IGE may contribute substantially to the heritable variation in the trait [33, 34]. Thus, a genetic model to accommodate both DGE and IGE explicitly is warranted. In our study, the sub-model for individual-level FI records did not consider IGE. However, the model for group-level FI records was not able to distinguish DGE and IGE, and hence, the additive genetic effect in the model of group records contained both DGE and IGE.

It is important to note that the group-level FI in this study was recorded on boars in a single nucleus herd and the individual FI was recorded on boars at the test station. Although in both cases, the pigs were group-housed under similar conditions and in pens of similar size, there are three differences between recording FI in the herd and at the test station. First, for the individual-level FI recorded at the test station, the experimental boars were selected across all DanBred nucleus herds and born in the same week; however, for the group-level FI recorded in the herd, the experimental boars were obtained only from boars born in the same herd. For the test station, one or two boars were selected among littermates from litters which had the highest range of selection index across all nucleus herds. Indeed, in our data, the start body weight of boars with individual-level FI was 30.5 kg (SD = 2.04 kg) while the start body weight of boars with group-level FI was 26.8 kg (SD = 2.50 kg) but the mean starting ages were similar, i.e., 78 days (SD = 4.6) and 78 days (SD = 5.9) for group- and individual-level boars, respectively. The use of different selection strategies for individual and group level recorded boars might reduce the genetic correlations, and also the differences in start weight and age can cause differences in FI which also decrease the genetic correlations between individual and group FI. Second, individual-level FI were recorded at the test station each time the boars visited the feeder, and a previous non-published study, which used criteria suggested by [35], showed that the individual feed records were very accurate across the whole growth period without any significant waste of feed. However, for the group-level FI in the herd, FI was recorded only twice during the experimental period and thus the group records could be affected by animals that drop out at the end of the testing interval. Furthermore, in group feeding the amount of wasted feed is unknown, and it can be hypothesized that wasted feed might increase the error variances and decrease the heritability for group FI boars. And third, for the group-level FI in the herd, each feeder was shared by two pens, whereas for the individual-level FI at the test station, each pen had its own feeder, thus the feeding group for group-level FI was larger than the feeding groups for individual-level FI. Furthermore, as group size increases, social interactions and competition may also increase, resulting in a negative response to selection [31].

The differences between test station and herds are evident, and thus may lead to re-ranking of animals across these two environments for the FI trait. Therefore, ranking animals based on (G)EBV estimated in one environment may change selection decisions that are defined in another environment. In particular, in the current Danbred breeding program, FI is generally evaluated based on data collected at the test station, whereas the breeding goal is defined at the level of the nucleus herds. This implies that ranking based on individual-level FI records may deviate from ranking based on group-level FI records in the herds. As a consequence, the effect of genotype-by-environment interaction can lower the efficiency of the breeding scheme if based on results from the test station environment [36]. Considering these differences, it may be necessary to consider the role and function of the test stations with caution.

The experiment in our study was set up to measure FI for pigs during their growth periods with bodyweight increasing from 30 to 100 kg. However, one issue with our study is that the group-level FI data were obtained only at two time points during the growth period. Thus, the final group-level FI data available for variance component estimation and genetic prediction were based on these two time points only, which may cause instability of the estimation of the residual variance. In addition, the estimated genetic correlations between group- and individual-level FI were relatively constant over time, but with large SE (Fig. 4), which can be attributed to the small size of the dataset used for group-level records. Thus, it might be worthwhile to collect more data across more time points to calculate group level FI, for further investigation.

## Conclusions

Using a bivariate random regression model, we estimated the genetic correlations between group- and individual-level FI and found low values that deviated significantly from 1 based on the current datasets. Our results support the hypothesis that group- and individual-level FI are two different traits, which indicates the presence of strong genotype-by-environment interaction between group- and individual-level FI. Gain in prediction accuracy for animals with group records was achieved by adding information from individual records via the use of bivariate models but this gain was limited due to the low genetic correlation between these two environments. The insights gained from our study may help current genetic evaluation strategies for FI to properly account for the differences in FI across different systems.

## Supplementary Information

**Additional file 1: Figure S1.** Scatter plot of Pearson’s correlations of (genomic) estimated breeding value ((G)EBV) based on full and reduced datasets ($\rho_{f,r}$) versus the absolute difference in (G)EBV between the full and reduced datasets ($d_{f,r}$) for all 323 groups for univariate PBLUP (panel A), bivariate PBLUP (panel B), univariate ssGBLUP (panel C), and bivariate ssGBLUP (panel D).
