# Supplementary figures and images for: Genetic parameters and genomic prediction for feed intake recorded at the group and individual level in different production systems for growing pigs
Source: Genet Sel Evol. 2021 Apr 8;53:33. doi: 10.1186/s12711-021-00624-3 (PMC8028714; doi:10.1186/s12711-021-00624-3)

A

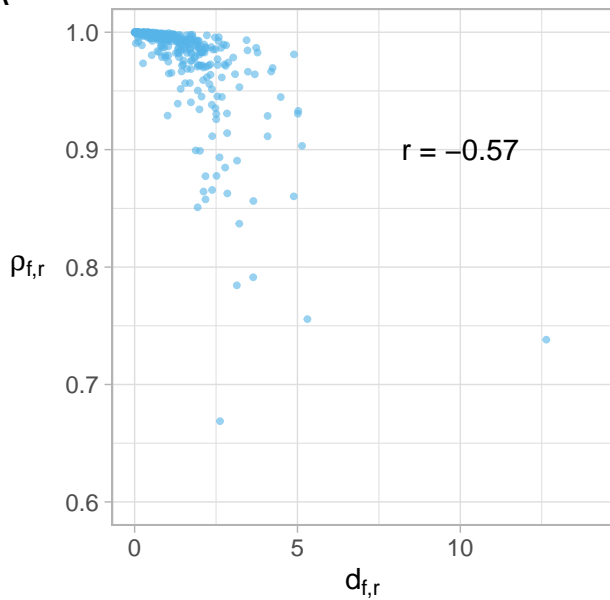

B

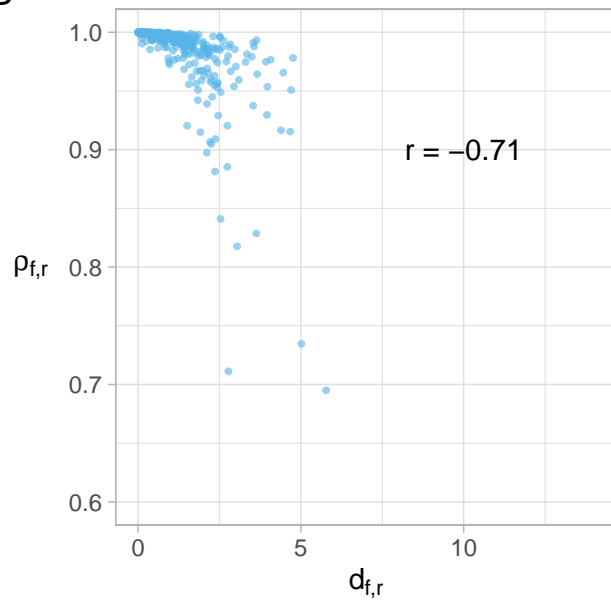

C

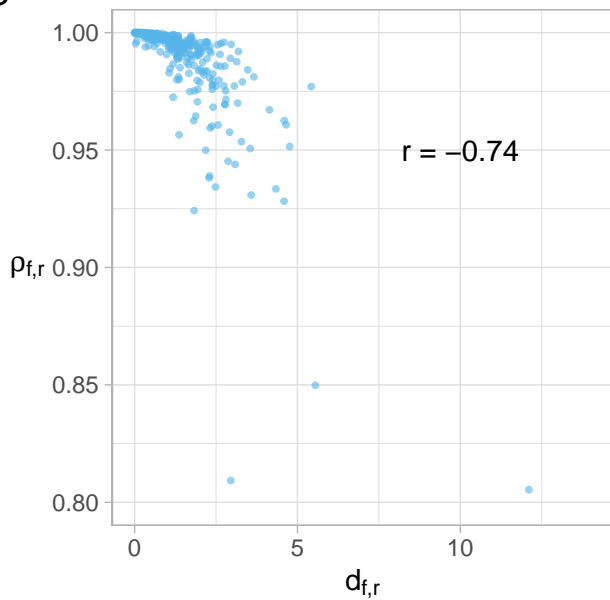

D

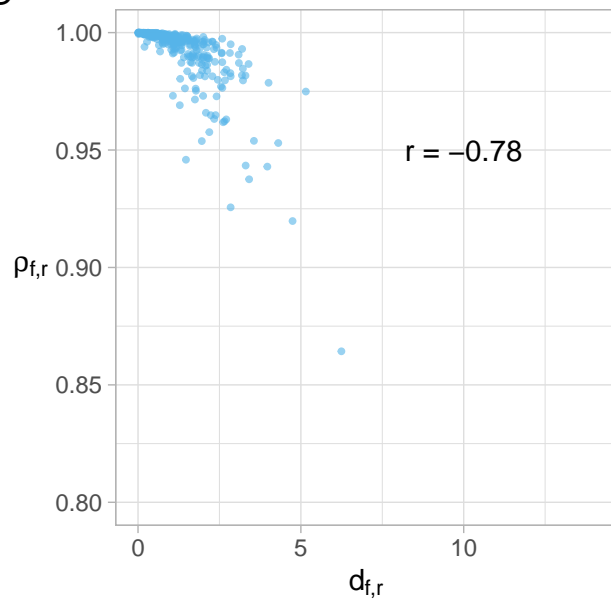

Supplement: Supplementary file 1 — Additional file 1: Figure S1. Scatter plot of Pearson’s correlations of (genomic) estimated breeding value ((G)EBV) based on full and reduced datasets ($\rho_{f,r}$ρf,r) versus the absolute difference in (G)EBV between the full and reduced datasets ($d_{f,r}$df,r) for all 323 groups for univariate PBLUP (panel A), bivariate PBLUP (panel B), univariate ssGBLUP (panel C), and bivariate ssGBLUP (panel D). [file 12711_2021_624_MOESM1_ESM.pdf]
